# Ferrostatin-1 and 3-Methyladenine Ameliorate Ferroptosis in OVA-Induced Asthma Model and in IL-13-Challenged BEAS-2B Cells

**DOI:** 10.1155/2022/9657933

**Published:** 2022-02-04

**Authors:** Nan Yang, Yunxiao Shang

**Affiliations:** Department of Pediatrics, Shengjing Hospital of China Medical University, Shenyang, Liaoning 110004, China

## Abstract

Ferroptosis was reported to be involved in the occurrence and development of asthma. However, the potential mechanism underlying the role of ferroptosis in asthma remains unclear. In this study, we established the mouse asthma model following the ovalbumin (OVA) method in C57BL/6 mice and the cell model with IL-13 induction in bronchial epithelial cells (BEAS-2B cells). Treatment of ferrostatin-1 (Ferr-1) and 3-methyladenine (3-MA) decreased iron deposition in IL-13-induced BEAS-2B cells and lung tissues of asthma mice, opposite to that in bronchoalveolar lavage fluid (BALF). Meanwhile, excessive lipid peroxidation asthma model *in vivo* and *in vitro* was alleviated by Ferr-1 or 3-MA treatment. In addition, Ferr-1 and 3-MA inhibited the expression of LC-3 in these cells and lung tissues of mice. Moreover, Ferr-1 and 3-MA also suppressed the production of inflammatory cytokines (IL-1*β*, IL-6, and TNF-*α*) and oxidative stress factors (ROS and MDA), while promoting the level of SOD, *in vivo* and *in vitro*. Furthermore, application of Ferr-1 exhibited a greater inhibitory effect on iron release and lipid peroxidation in IL-13-induced BEAS-2B cells and asthma mice than 3-MA, accompanied with a weaker effect on ferritinophagy than 3-MA. Collectively, Ferr-1 and 3-MA ameliorated asthma *in vivo* and *in vitro* through inhibiting ferroptosis, providing a new strategy for the clinical treatment of asthma.

## 1. Introduction

Asthma is a chronic airway inflammation which is associated with multiple cells and cellular components [1]. This inflammation often induces adverse respiratory reactions and symptoms such as shortness of breath, chest tightness, and cough [2]. Furthermore, asthma is a common chronic respiratory disease with a high incidence in all age groups [3]. In recent years, the incidence of asthma has been increasing around the world. Several overlapping factors such as inhaled allergens, air pollution, respiratory virus infections, and genetics could trigger the initiation of asthma [4]. In addition, the ferroptotic and autophagic molecular mechanisms of the early pathophysiology of asthma are still unclear to develop new clinical therapies.

Ferroptosis is an iron-dependent form of cell death [5]. The occurrence of ferroptosis is mainly due to the excessive accumulation of reactive oxygen species (ROS) related to iron ions in the cells and the attenuation scavenging effect of glutathione peroxidase 4 (GPX-4). These can lead to the formation of lipid oxides [6], and the homeostasis of the production and degradation of lipid oxide is also imbalanced, resulting in the disability of clearing the excessive accumulation of lipid oxides in time, which will finally cause cell death [7]. Iron in cells is often in the form of ferritin, such as ferritin light chain (FTL) and ferritin heavy chain (FTH1), and iron response element binding protein 2 (IREB2) plays a critical role in the formation of ferritin [8]. There is a study revealing that airway inflammation, lipid peroxidation and ROS levels were elevated in asthma, accompanied with the occurrence of ferroptosis, indicating that ferroptosis was involved in the pathogenesis of allerigic asthma [9].

The occurrence of inflammation of respiratory epithelial cells was a common pathogenesis of the asthma [10]. The study suggested that the expression of interleukin- (IL-) 13 could activate the expression of members of the lipoxygenase family (ALOX15 and ALOX5) and therefore aggravated the oxidation of polyunsaturated fatty acid phosphatidylerhanolamine (PUFA-PE), thereby inducing the occurrence of ferroptosis [11]. However, the production of GPX-4 could induce the production of reduced glutathione (GSH), and the releasing of GSH relieved the peroxidate of these cells, so as to avoid ferroptosis [12]. However, whether GPX-4 and GSH could alleviate asthma by relieving ferroptosis is unclear.

In this study, we established an asthma model in vivo and in vitro and used ferrostatin-1 (Ferr-1), a synthetic antioxidant that has been recognized as an inhibitor of ferroptosis [13, 14], and 3-methyladenine (3-MA), an inhibitor of phosphatidylinositol 3-kinases (PI3K) that is commonly used as autophagy inhibitor [15, 16], for treatment, to explore the molecular mechanism of ferroptosis during asthma. This study may provide a novel therapeutic strategy for the treatment of asthma.

## 2. Materials and Methods

### 2.1. Animal Assays

First, male C57BL/6 mice were obtained from Shanghai Institute of Zoology, Chinese Academy of Sciences, and housed in a temperature- and humidity-controlled room under a 12/12 h light/dark cycle with free access to water and food. All animal studies were approved by the ethics review committee of Shengjing Hospital of China Medical University.

**Figure 1:**
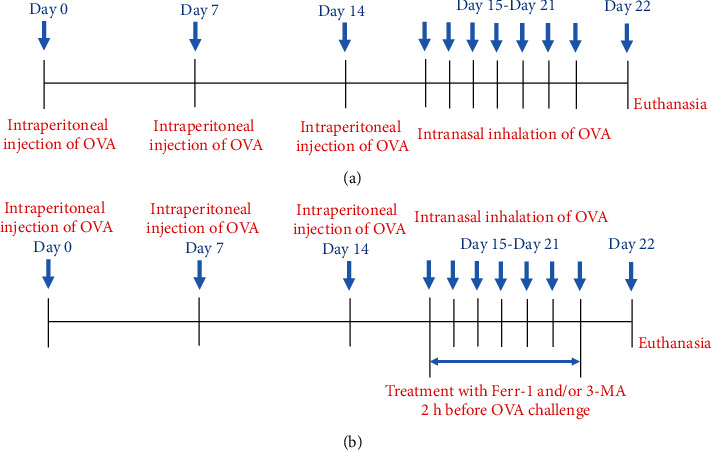
Experimental scheme. (a) Experimental scheme for OVA-induced asthma. (b) Experimental scheme for OVA-induced asthma and treatment with Ferr-1 and/or 3-MA. OVA: ovalbumin; Ferr-1: ferrostatin-1; 3-MA: 3-methyladenine.

The *in vivo* experiments consist of two parts (Figures 1(a) and 1(b)). For part 1, 10 mice were stochastically divided into 2 groups: the control group and model group. The animal model of asthma was established following the ovalbumin (OVA) method. In brief, the mice of the model group were sensitized by intraperitoneal injection of 1 mg OVA/40 mg Al(OH)_3_ gel in 0.2 ml phosphate buffer saline (PBS; once a week) for 3 times. On the next day, after the last time of OVA injection, the mice were then challenged by intranasal inhalations with 5% OVA for a week. Mice in the control group received the same schedule for sensitization and were administrated with an equivalent amount of 0.9% sterile saline instead of OVA.

For part 2, 25 mice were randomly divided into 5 groups: the control group, model group, Ferr-1 group, 3-MA group, and Ferr-1+3-MA group. The mice in the control and model groups were proceeded as described above. The mice of the Ferr-1 group were treated with the Ferr-1 (2.5 *μ*mol/kg, dissolved in dimethylsulfoxide (DMSO); Selleck, USA) by the intraperitoneal injection 2 h prior to OVA challenge. The dose of Ferr-1 was selected according to previous studies [17, 18]. The mice of the 3-MA group were intraperitoneally injected with the 3-MA (15 mg/kg, dissolved in DMSO; Sigma-Aldrich, USA) 2 h prior to OVA challenge [19, 20]. The mice of Ferr-1+3-MA group were treated with both Ferr-1 and 3-MA. Finally, these mice were executed to euthanasia. Subsequently, PBS was used for the lavage of the bronchus. Then, the PBS was collected as the bronchoalveolar lavage fluid (BALF) for the next research. In addition, a part of lung tissues were fixed in 4% formaldehyde, and the remaining lung tissues were stored at -80°C for further experiments.

### 2.2. Cell Culture and Treatment

Bronchial epithelial cell line (BEAS-2B cells) was purchased from American Type Culture Collection (ATCC; Manassas, VA, USA). These cells were cultured with RPMI-1640 medium (Hyclone, USA) supplemented with 10% fetal bovine serum (Gibco, USA). These cells were cultured in the 37°C humid atmosphere supplemented with 5% CO_2_. These cells were cultured in air-liquid interface (ALI) as previously described [12] and treated with the IL-13 (2 ng/ml, 10 ng/ml, and 50 ng/ml, Thermo Fisher Scientific, USA) to mimic asthma injury *in vitro*. 3-MA (5 *μ*g/ml) and Ferr-1 (3 *μ*g/ml) were also used for the treatment of these cells.

### 2.3. Hematoxylin Eosin Staining and Perls Staining

The lung tissues was fixed with 10% neutral buffered formalin, dehydrated with graded alcohol, embedded with paraffin, and then cut into 3-5 mm slices. Next, the slices were incubated with the hematoxylin staining solution (Beyotime, China) for 15 minutes. Then, the tissues were washed with PBS and stained with eosin for 15 minutes. Finally, these tissues were observed under the light microscope (Leica, Germany). The rest tissues were also stained with Prussian blue (Sigma-Aldrich, St. Louis, MO) for 30 minutes after the fixation of these tissues. And these tissues were also observed under the light microscope (Leica, Germany) after staining.

### 2.4. Detection of Ferric Ion

Commercial kits (C0165S and C0166S, Beyotime, China) were applied for the detection of ferric ions in lung tissues and BEAS-2B cells. The operation of this experiment was following the instruction.

### 2.5. Immunohistochemical Staining

We used paraffin to embed the lung tissue and cut the tissue into thin slices. Next, xylene was used for the dewaxing of these tissues. After that, these tissues were incubated with H_2_O_2_ and stewed in the citrate buffer solution to expose the antigen sites. Then, these tissues were blocked with BSA (Beyotime, China) and incubated with the primary antibody against LC-3 (ab48394, Abcam) at 4°C overnight. On the second day, the tissues were incubated with the secondary antibody (Goat Anti-Rabbit IgG H&L, ab205718, Abcam) at room temperature for 2 hours. Then, these tissues were stained with the hematoxylin solution (Beyotime, China). Finally, the expression of LC-3 in these tissues was observed under the microscope (Olympus, Japan).

### 2.6. Immunofluorescence

BEAS-2B cells were plated on the glass slide. These cells were fixed with 4% paraformaldehyde and incubated with 0.5% Triton X-100 (Beyotime, China) to enhance the permeability of the cell membranes. Then, 5% skim milk powder solution was used for the block of these cells. And these cells were incubated with the primary antibody against LC-3 (ab48394, Abcam) at 4°C overnight. After that, these cells were incubated with the secondary antibody (Goat Anti-Rabbit IgG, ab150077, Abcam) in the dark room. Finally, DAPI was used for the mark of the cell nucleus and the fluorescence was observed under the confocal microscope (Olympus, Japan).

### 2.7. ELISA Assays

Human IL-6 ELISA kit (ab178013, Abcam), human TNF-*α* ELISA kit (ab181421, Abcam), and human IL-1*β* ELISA kit (ab229384, Abcam) were used for the detection of these factors in culture medium of BEAS-2B cells. In addition, mouse IL-1*β* ELISA kit (ab197742, Abcam), mouse IL-6 ELISA kit (ab222503, Abcam), and mouse TNF-*α* ELISA kit (ab208348, Abcam) were applied for the detection of the expression of these factors in BALF and lung tissues of mice. In addition, mouse FTL ELISA kit (ab157713, Abcam) and mouse FTH1 ELISA kit (ab65080, Abcam) were applied for the detection of the expression of these proteins in BALF and lung tissues of mice. The operation was following the instruction.

### 2.8. The Detection of Reduced Glutathione (GSH) and Oxidized Glutathione (GSSG)

The commercial kits (Beyotime, China) were used for the detection of GSH and GSSG in lung tissues and BEAS-2B cells. The operation was following the instruction.

### 2.9. Detection of ROS, MDA, and SOD

The production of SOD and MDA in lung tissues and BEAS-2B cells were determined with their commercial kits from Beyotime (S0088 and C0017, Shanghai, China). All operations of this assay were following the instruction. The ROS level was detected using its commercial kit from Shanghai Jianglai Biological Technology Co., Ltd. (Shanghai, China).

### 2.10. Cell Counting Kit-8 (CCK-8) Assay

BEAS-2B cells were plated into 96 plates. After the incubation for 24, 48, and 72 h, respectively, CCK-8 (Dojindo, Japan) was diluted and added into 96 plates. Next, these cells were incubated in the incubator for 2 hours. Finally, the absorbance at 450 nm of these cells was determined with the spectrophotometer.

### 2.11. Western Blotting

Total protein was extracted with RIPA buffer (Beyotime, China). Next, the concentration of these samples was determined with the BCA method (Beyotime, China). Then, these proteins were separated by the 10% SDS-PAGE gel (Beyotime, China). After that, these proteins were transferred to the polyvinylidene fluoride (PVDF) membranes (Millipore, USA). These membranes were blocked with 5% defatted milk at room temperature for 2 h and incubated with the primary antibodies at 4°C overnight. The primary antibodies used in this research were GPX-4 (ab125066, Abcam), solute carrier family 7 member 11 (SLC7A11; ab37185, Abcam), solute carrier family 3 member 2 (SLC3A2; sc-390154, Santa Cruz), arachidonate-5-lipoxygenase (ALOX5; ab169755, Abcam), autophagy-related 5 (ATG5; ab108327, Abcam), ATG7 (ab133528, Abcam), nuclear receptor coactivator 4 (NCOA4; H00008031-M04, Novus), transferrin receptor (TFR1, ab84036, Abcam), divalent metal transporter 1 (DMT-1; ab222895, Abcam), and GAPDH (ab9485, Abcam). On the second day, these membranes were washed with PBST and incubated with the secondary antibody (Goat anti-rabbit IgG, ab150077, Abcam) for 2 hours. Finally, the bands were developed with enhanced chemiluminescence (ECL) substrates (Millipore, USA).

### 2.12. Statistical Analysis

All data in this research were analyzed with the GraphPad Prism 8.0 (GraphPad Software, Inc.). The experiments of this study were repeated for three times, and the data in this research were displayed as mean ± SD. The comparison between diverse groups was performed with Student's *t*-test or one-way ANOVA test. The difference was considered as statistical significance until the value of *p* was less than 0.05.

## 3. Results

### 3.1. The Ferroptosis Occurred in the Lung Tissues of Mice during the Development of Asthma

**Figure 2:**
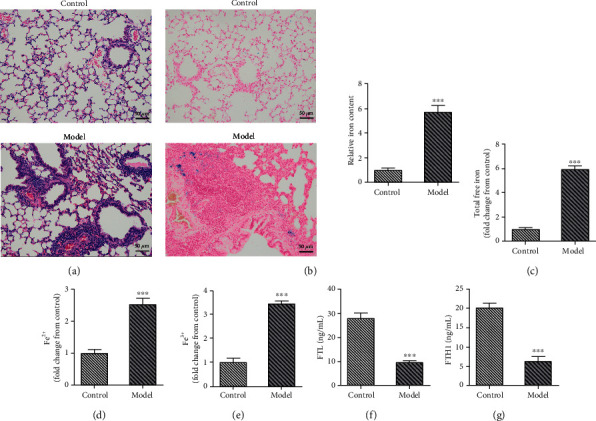
Asthma-induced ferroptosis. (a) H&E staining was used for the detection of the injury of lung tissues. (b) Perls staining was used for the detection of iron in lung tissues. (c–e) The levels of free iron ions were detected with commercial kits. (f, g) The expression of ferroprotein, including FTL and FTH1, was detected with ELISA. ^∗∗∗^*p* < 0.001. FTL: ferritin light chain; FTH1: ferritin heavy chain.

In this part, we established the asthma model mice and collected the BALF and lung tissues after the sacrifice of these mice. As shown in Figure 2(a), a severe pathological injury of lung tissues was observed in asthma mice. The results (Figures 2(b)–2(e)) of Prussian blue staining and iron ion detection revealed an iron deposition in the lung tissues of asthma mice, compared to the mice of the control group. In addition, we found that the expression of FTL and FTH1 was suppressed in the lung tissues of asthma model mice (Figures 2(f) and 2(g)). All these results suggested that the ferroptosis was activated during the development of asthma.

### 3.2. Treatment of Ferr-1 and/or 3-MA Relieved Asthma-Induced Damage of Lung Tissues of Mice

**Figure 3:**
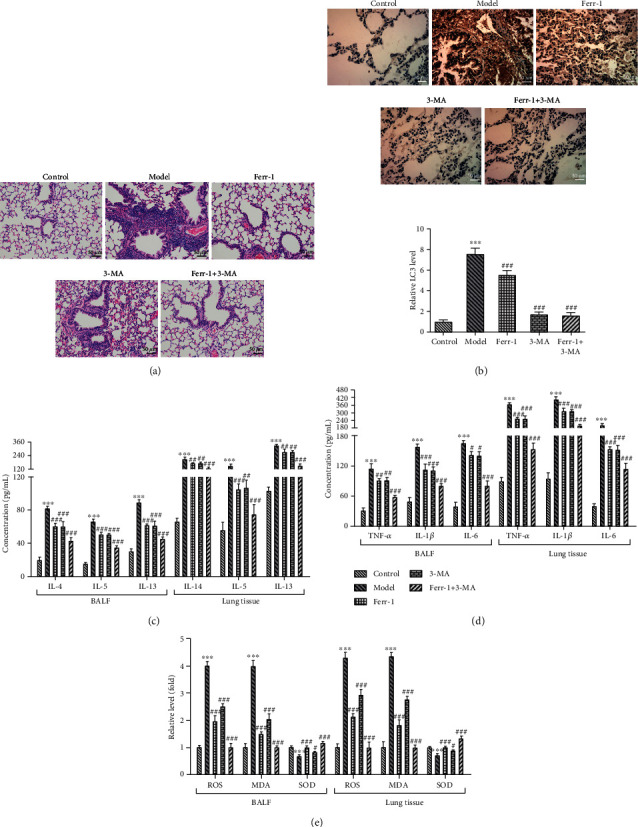
Ferr-1 and 3-MA alleviated the inflammation and oxidative damage of lung tissues in asthma mice. (a) H&E staining was used for the detection of the damage of lung tissues. (b) The expression of LC-3 in lung tissues was detected with immunohistochemistry. (c) The levels of IL-4, IL-5, and IL-13 in lung tissues and BALF were determined with ELISA. (d) The levels of IL-1*β*, IL-6, and TNF-*α* in lung tissues and BALF were determined with ELISA. (e) The levels of ROS, SOD, and MDA in lung tissues and BALF were detected with commercial kits. ^∗∗∗^*p* < 0.001 vs. the control; ^#^*p* < 0.05, ^##^*p* < 0.01, and ^###^*p* < 0.001 vs. the Model. Ferr-1: ferrostatin-1; 3-MA: 3-methyladenine; BALF: bronchoalveolar lavage fluid.

Next, Ferr-1 and 3-MA were used for the treatment of asthma mice. Results (Figure 3(a)) of H&E staining showed that the application of Ferr-1 and 3-MA alleviated the pathological injury of lung tissues of asthma mice. Meanwhile, a high expression of LC3, the marked protein of autophagy, was identified in lung tissues of asthma mice, which was then partly abolished by Ferr-1 and/or 3-MA treatment (Figure 3(b)). In addition, the concentrations of IL-4, IL-5, and IL-13, as well as IL-1*β*, IL-6, and TNF-*α*, were greatly elevated in BALF and lung tissues, especially in lung tissues, of asthma mice, which were suppressed after the treatment of Ferr-1 and/or 3-MA (Figures 3(c) and 3(d)). The commercial kits were also used for the detection of ROS, SOD, and MDA in BALF and lung tissues of these mice. What is more, the downregulated SOD and upregulated ROS and MDA in BALF of asthma mice were obviously reversed by Ferr-1 and/or 3-MA treatment. The levels of ROS, MDA, and SOD in the lung tissue had the same trendline of change with those in BALF (Figure 3(e)).

### 3.3. Treatment of Ferr-1 and/or 3-MA Relieved Iron Accumulation in Lung Tissues in Asthma Mice

**Figure 4:**
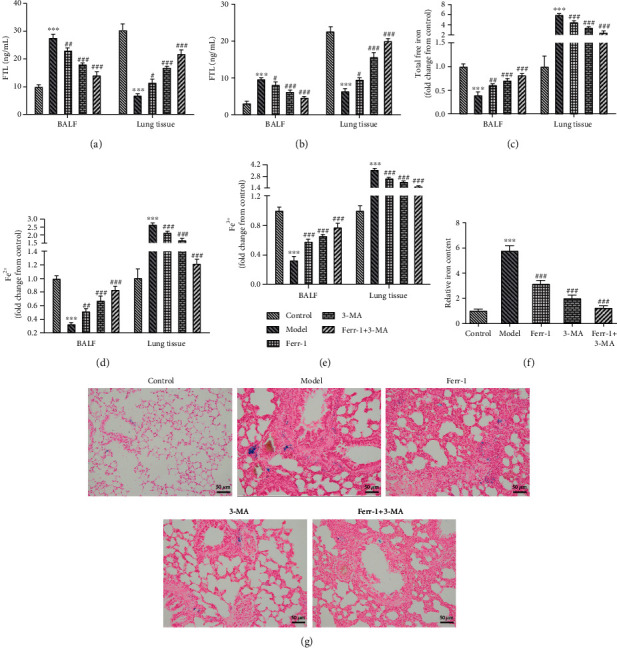
Ferr-1 and 3-MA relieved the accumulation of free iron ions in lung tissues in asthma mice. (a) The levels of FTL in BALF were determined with ELISA. (b) The levels of FTH1 in BALF were determined with ELISA. (c–e) The levels of free iron ions in BALF and lung tissues were detected with commercial kits. (f, g) Perls staining was used for the detection of Fe in lung tissues. ^∗∗∗^*p* < 0.001 vs. the control; ^#^*p* < 0.05, ^##^*p* < 0.01, and ^###^*p* < 0.001 vs. the model. Ferr-1: ferrostatin-1; 3-MA: 3-methyladenine; FTL: ferritin light chain; FTH1: ferritin heavy chain; BALF: bronchoalveolar lavage fluid.

Next, we explored the specific regulatory role of Ferr-1 and 3-MA in asthma. As shown in Figures 4(a) and 4(b), the levels of FTL and FTL1 in BALF were increased after the occurrence of asthma while decreased after the Ferr-1 and/or 3-MA treatment. On the contrary, the levels of FTL and FTL1 in lung tissues were decreased in asthma mice but increased after Ferr-1 and/or 3-MA treatment. Meanwhile, as shown in Figures 4(c)–4(e), the levels of free iron in BALF were decreased during the development of asthma and increased after the treatment of Ferr-1 and/or 3-MA, which was opposite to those in lung tissues. Similarly, the results of Perls staining also revealed an accumulation of iron in lung tissue of asthma mice, while Ferr-1 and 3-MA treatment relieved this accumulation (Figures 4(f) and 4(g)). These results suggested that Ferr-1 and/or 3-MA treatment reduced iron deposition in lung tissues of asthma mice.

### 3.4. Treatment of Ferr-1 and/or 3-MA Alleviated Lipid Peroxidation and Ferritinophagy of Lung Tissues in Asthma Mice

**Figure 5:**
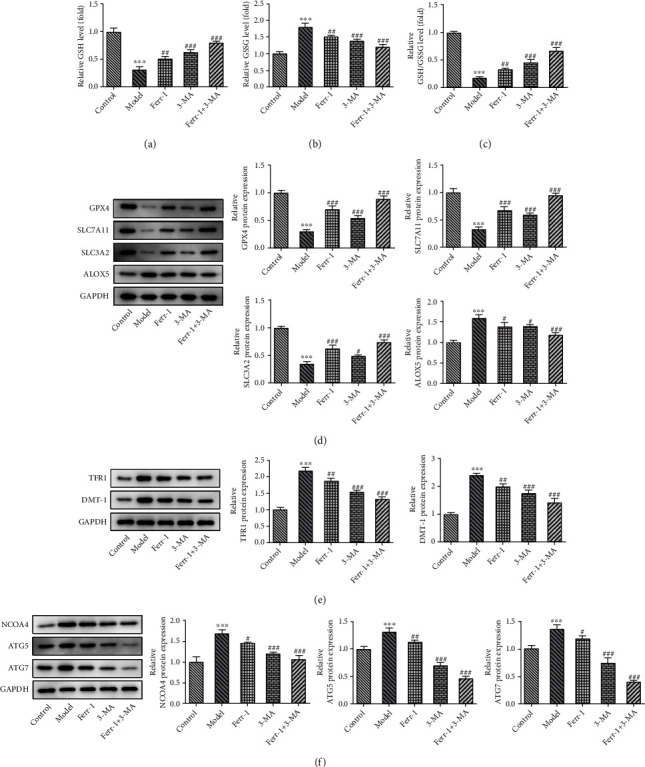
Ferr-1 and 3-MA relieved lipid peroxidation in lung tissues in asthma mice. (a–c) The level of GSH and GSSG was determined with the commercial kits. (d) The expression of GPX4, SLC7A11, SLC3A2, and ALOX5 in lung tissues was determined with western blotting. (e) The expression of NCOA4, TFR1, and DMT-1 in lung tissues was detected with western blotting. (f) The expression of ATG5 and ATG7 in lung tissues was determined with western blotting. ^∗∗∗^*p* < 0.001 vs. the control; ^#^*p* < 0.05, ^##^*p* < 0.01, and ^###^*p* < 0.001 vs. the model. Ferr-1: ferrostatin-1; 3-MA: 3-methyladenine; GSH: reduced glutathione; GSSG: oxidized glutathione; GPX4: glutathione peroxidase 4; SLC7A11: solute carrier family 7 member 11; SLC3A2: solute carrier family 3 member 2; ALOX5: arachidonate-5-lipoxygenase; NCOA4: nuclear receptor coactivator 4; TFR1: transferrin receptor; DMT-1: divalent metal transporter 1; ATG5: autophagy-related 5; ATG7: autophagy-related 7.

In addition, we also explored the lipid peroxidation and ferritinophagy, which were critical regulatory mechanisms underlying the initiation of ferroptosis, in Ferr-1- and 3-MA-treated asthma mice. As exhibited in Figures 5(a)–5(c), the treatment of Ferr-1 and 3-MA promoted the production of GSH and restricted the production of GSSG in the lung tissues of asthma mice. Meanwhile, we found a downregulated expression of antioxidant proteins (SLC7A11, SLC3A2, and GPX-4) and an upregulated expression of ALOX5, which was related to lipid peroxides-induced ferroptosis, in the lung tissues of asthma mice; however, the expression of these antioxidant proteins was enhanced, while the levels of ALOX5 were suppressed after the treatment of Ferr-1 and 3-MA (Figure 5(d)). Moreover, a previous report pointed out that the increased TFR1 and DMT1 expression were linked with elevated ferroptosis and severe asthma [21], which was also verified in the present study, but Ferr-1 and/or 3-MA treatment could greatly reduce the protein expression of TFR1 and DMT1 (Figure 5(e)). Furthermore, the expression of ferritinophagy-related proteins (NCOA4, ATG-5, and ATG-7) in the lung tissues was elevated upon the occurrence of asthma but was then repressed after the treatment of Ferr-1 and 3-MA (Figure 5(f)).

### 3.5. IL-13 Induced Inflammatory and Oxidative Damage and Induced Ferroptosis of Bronchial Epithelial Cells

**Figure 6:**
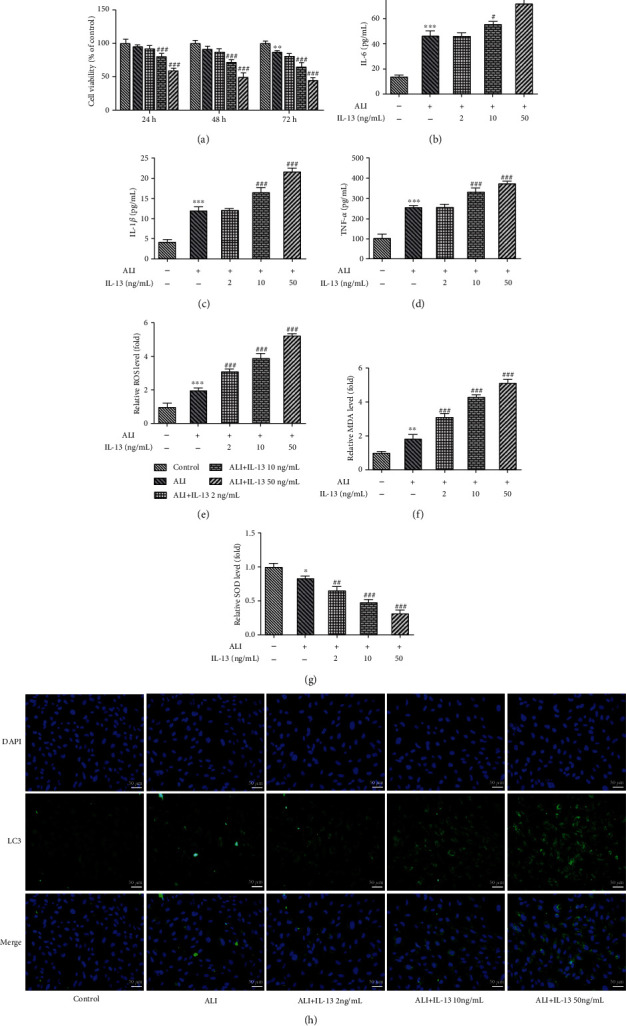
ALI and IL-13 induced the damage of bronchial epithelial cells. (a) CCK-8 was performed for the detection of the viability of these cells. (b–d) The levels of IL-1*β*, IL-6, and TNF-*α* in these cells were determined with ELISA. (e–g) The levels of ROS, SOD, and MDA in these cells were detected with commercial kits. (h) The expression of LC-3 in these cells was detected with immunofluorescence. ^∗^*p* < 0.05, ^∗∗^*p* < 0.01, and ^∗∗∗^*p* < 0.001 vs. the control; ^#^*p* < 0.05, ^##^*p* < 0.01, and ^###^*p* < 0.001 vs. ALI. ALI: air-liquid interface; CCK-8: cell counting kit-8; ROS: reactive oxygen species; SOD: superoxide dismutase; MDA: malonaldehyde.

**Figure 7:**
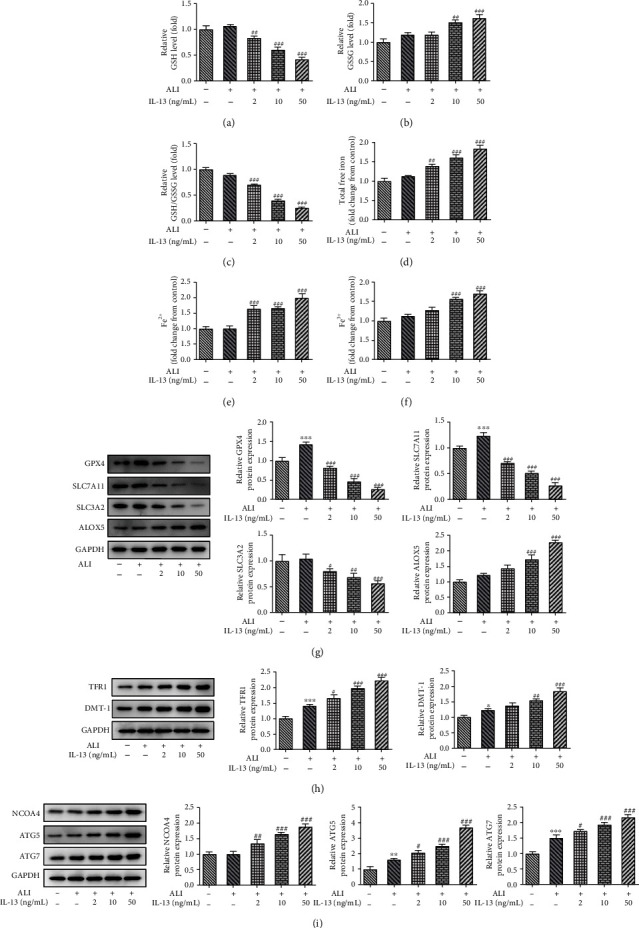
ALI and IL-13 induced ferroptosis in bronchial epithelial cells (BEAS-2B cells). (a–c) The level of GSH and GSSG was determined with the commercial kits. (d–f) The levels of free iron ions in these cells were detected with commercial kits. (g) The expression of GPX4, SLC7A11, SLC3A2, and ALOX5 in these cells was determined with western blotting. (h) The expression of NCOA4, TFR1, and DMT-1 in these cells was detected with western blotting. (i) The expression of ATG5 and ATG7 in these cells was determined with western blotting. ^∗^*p* < 0.05, ^∗∗^*p* < 0.01, and ^∗∗∗^*p* < 0.001 vs. the control; ^#^*p* < 0.05, ^##^*p* < 0.01, and ^###^*p* < 0.001 vs. ALI. ALI: air-liquid interface; GSH: reduced glutathione; GSSG: oxidized glutathione; GPX4: glutathione peroxidase 4; SLC7A11: solute carrier family 7 member 11; SLC3A2: solute carrier family 3 member 2; ALOX5: arachidonate-5-lipoxygenase; NCOA4: nuclear receptor coactivator 4; TFR1: transferrin receptor; DMT-1: divalent metal transporter 1; ATG5: autophagy-related 5; ATG7: autophagy-related 7.

To further concentrate on the regulatory mechanism of Ferr-1 and 3-MA in asthma, bronchial epithelial cells (BEAS-2B cells) were applied and stimulated with IL-13 to mimic asthma *in vitro*. Firstly, stimulation of IL-13 induced the decreasing of the viability of BEAS-2B cells dose dependently (Figure 6(a)). The production of IL-1*β*, IL-6, and TNF-*α* was also hugely elevated after the stimulation of IL-13 at 10 ng/ml and 50 ng/ml (Figures 6(b)–6(d)). Moreover, stimulation of IL-13 also enhanced the production of ROS and MDA, while repressing the activity of SOD in BEAS-2B cells (Figures 6(e)–6(g)). In addition, the results (Figure 6(h)) of immunofluorescence also showed that stimulation of IL-13 enhanced the expression of LC-3. Next, the reduced GSH/GSSG ratio and the elevated free iron upon IL-13 stimulation revealed that IL-13 induced oxidative damage and iron deposition in BEAS-2B cells (Figures 7(a)–7(f)), which was also evidenced by the downregulated protein expression of GPX4, SCL7A11, and SLC3A2 and the upregulated expression of ALOX5 upon IL-13 stimulation (Figure 7(g)), suggesting that IL-13 triggered the initiation of ferroptosis in BEAS-2B cells. Consistently, IL-13 also promoted the expression of ferroptosis-related proteins (TFR1, DMT1) and ferritinophagy-related proteins (NCOA4, ATG-5, and ATG-7) in BEAS-2B cells (Figures 7(h) and 7(i)).

### 3.6. Ferr-1 or 3-MA Relieved the IL-13-Induced Inflammation, Oxidative Damage, and Ferroptosis of Bronchial Epithelial Cells

**Figure 8:**
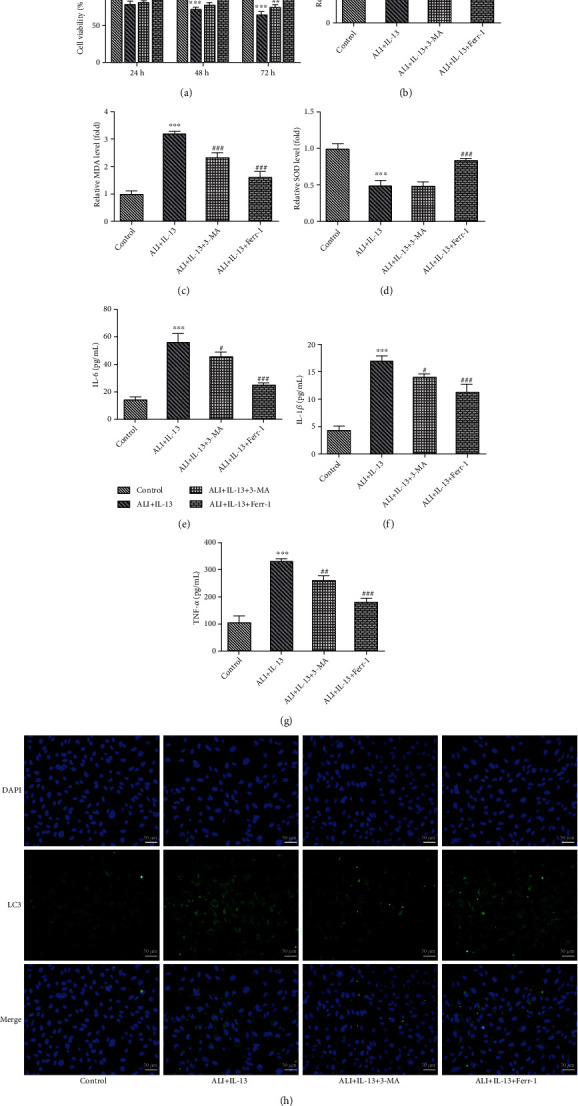
Ferr-1 and 3-MA relieved the inflammation and oxidative damage of IL-13-induced BEAS-2B cells. (a) CCK-8 was performed for the detection of the viability of these cells. (b–d) The levels of ROS, SOD, and MDA in these cells were detected with commercial kits. (e–g) The levels of IL-1*β*, IL-6, and TNF-*α* in these cells were determined with ELISA. (h) The expression of LC-3 in these cells was detected with immunofluorescence. ^∗∗∗^*p* < 0.001 vs. the control; ^#^*p* < 0.05, ^##^*p* < 0.01, and ^###^*p* < 0.001 vs. ALI+IL-13. Ferr-1: ferrostatin-1; 3-MA: 3-methyladenine; ALI: air-liquid interface; CCK-8: cell counting kit-8; ROS: reactive oxygen species; SOD: superoxide dismutase; MDA: malonaldehyde.

**Figure 9:**
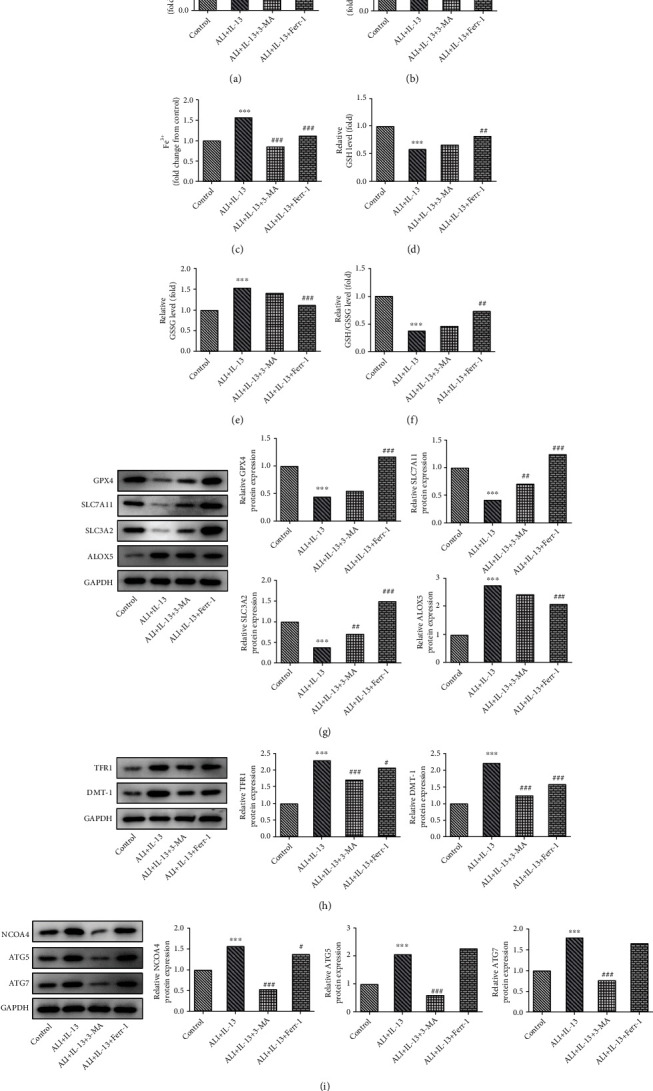
Ferr-1 and 3-MA relieved iron deposition, lipid peroxidation, and ferritinophagy in IL-13-induced BEAS-2B cells. (a–c) The levels of free iron ions in these cells were detected with commercial kits. (d–f) The ratio of GSH/GSSG was determined with the commercial kits. (g) The expression of GPX4, SLC7A11, SLC3A2, and ALOX5 in these cells was determined with western blotting. (h) The expression of NCOA4, TFR1, and DMT-1 in these cells was detected with western blotting. (i) The expression of ATG5 and ATG7 in these cells was determined with western blotting. ^∗∗∗^*p* < 0.001 vs. the control; ^#^*p* < 0.05, ^##^*p* < 0.01, and ^###^*p* < 0.001 vs. ALI+IL-13. Ferr-1: ferrostatin-1; 3-MA: 3-methyladenine; ALI: air-liquid interface; GSH: reduced glutathione; GSSG: oxidized glutathione; GPX4: glutathione peroxidase 4; SLC7A11: solute carrier family 7 member 11; SLC3A2: solute carrier family 3 member 2; ALOX5: arachidonate-5-lipoxygenase; NCOA4: nuclear receptor coactivator 4; TFR1: transferrin receptor; DMT-1: divalent metal transporter 1; ATG5: autophagy-related 5; ATG7: autophagy-related 7.

Finally, due to a severe damage to cell viability of IL-13 at 50 ng/ml, IL-13 at 10 ng/ml was selected for the subsequent experiments. In addition, Ferr-1 and 3-MA were applied for treatment *in vitro*. As shown in Figure 8(a), the treatment of Ferr-1 or 3-MA recovered the viability of BEAS-2B cells induced by IL-13. Ferr-1 or 3-MA also suppressed the production of ROS and MDA while enhancing the releasing of SOD (Figures 8(b)–8(d)). Similarly, the treatment of Ferr-1 or 3-MA suppressed the production of IL-1*β*, IL-6, and TNF-*α* in IL-13-stimulated BEAS-2B cells (Figures 8(e)–8(g)). Moreover, we also found that treatment of Ferr-1 and 3-MA inhibited the expression of LC-3 in these cells (Figure 8(h)). In addition, the results also showed that the free iron ion was decreased after the treatment of Ferr-1 or 3-MA (Figures 9(a)–9(c)). The treatment of Ferr-1 also rescued the production of GSH while inhibiting the releasing of GSSG in IL-13-stimulated BEAS-2B cells, but 3-MA did not affect the level of GSH and GSSG in IL-13-stimulated BEAS-2B cells (Figures 9(d)–9(f)). Moreover, Ferr-1 treatment remarkably rescued the expression of SLC7A11, SLC3A2, GPX-4, and ALOX5, but 3-MA only rescued the expression of SLC7A11 and SLC3A2 in IL-13-stimulated BEAS-2B cells (Figure 9(g)). The levels of TFR1 and DMT-1 in these cells were further inhibited after the treatment of Ferr-1 or 3-MA (Figure 9(h)). Furthermore, in terms of ferritinophagy-related proteins (NCOA4, ATG-5, and ATG-7), 3-MA exhibited a significantly inhibitory effect on IL-13-mediated ferritinophagy, but Ferr-1 did not affect it well (Figure 9(i)).

## 4. Discussion

Asthma is a widespread respiratory disease. The occurrence and development of asthma affect the health of many people. At present, the main clinical therapy is the use of glucocorticoids or leukotriene receptor antagonists to relieve the symptoms of asthma patients [22]. While traditional anti-inflammatory drugs could induce many side effects and damage human health, new treatment options were needed to further improve safety and effectiveness [23]. Therefore, we need to further study the molecular mechanisms of the occurrence and development of asthma to develop the latest treatment options. This study evaluated the initiation of ferroptosis in the pathogenesis of asthma *in vivo* and *in vitro*, as well as the potential regulatory mechanism.

Ferroptosis usually refers to the process of excessive lipid peroxide caused by the accumulation of a large amount of free iron in the cell, which eventually induces cell death [24]. As aforementioned, GSH, a major antioxidant that acts as a reducing substrate of GPX4 to mitigate the accumulation of ROS, can reduce and alleviate the accumulation of lipid peroxides in cells [25]. Heterodimeric cystine/glutamate antiporter system Xc^−^ (mainly consisting of SLC7A11 and SLC3A2) mainly focuses on taking cystine into cells and reducing it to cysteine to synthesize GSH, thereby protecting cells from oxidative damage. Therefore, regardless of the abnormal function of the Xc^−^-system, the abnormal GSH synthesis, or the decreased GPX4 activity caused by various factors, it could induce the hindering of the reduction of lipid peroxides and finally led to the ferroptosis of cells [26]. Currently, ferroptosis has been reported to be closely involved in and participated into human biological processes and multiple human diseases, including cancer, lung injury, and kidney diseases [13, 27, 28]. Nevertheless, there has been very few evidence regarding ferroptosis in asthma. A previous study reported that ferroptosis-inducing agents (FINs) could induce ferroptosis-like cell death of eosinophils, which was hindered by GSH, suggesting an involvement of ferroptosis in airway inflammation, and restraining of ferroptosis might alleviate allergic airway inflammation [29]. A latest study by Tang et al. reported environmental allergens house dust mite- (HDM-) induced asthma was associated with ferroptosis in the lungs, as downregulation of SLC7A11, GSH, GPX4, and ROS was detected in lung tissues of HDM-induced asthma mice [9]. In the present study, we found a decreased ratio of GSH/GSSG, as well as an elevated level of ROS and MDA, which reflected the oxidative damage of cells or tissues [30]. Consistently, the expression of SLC7A11, SLC3A2, and GPX-4 was also inhibited, while the level of ALOX5 was enhanced in lung tissues of asthma mice or IL-13-stimulated BEAS-2B cells, further demonstrating the initiation of ferroptosis during the development of asthma. Nevertheless, the application of Ferr-1 relieved above changes induced by asthma. These results implied that excessive production of lipid peroxide led to the ferroptosis of lung tissues and bronchial epithelial cells, and the application of ferroptosis inhibitor exerted a protective role in asthma by alleviating oxidative damage.

In addition to the dysregulation of lipid metabolism, the initiation and progression of ferroptosis are also influenced by iron levels. Excessive iron deposition leads to lipid peroxidation, which results in excessive production of lipid peroxide and triggering free radicals via Fenton reaction, eventually inducing ferroptosis [17, 31]. The relationship between iron level and asthma has been supported by previous evidence, which reported that cell-free iron level was decreased in BALF of severe or mild-moderate asthma patients. Meanwhile, the expression level of DMT1 and TFR1 in lung tissues of asthma patients was greatly increased, which was correlated with reduced lung function [21]. In line with the previous findings, a reduced free iron in BALF and an increased expression of TFR1 and DMT-1 in lung tissues were also found in asthma mice in the this study. Accordingly, FTL and FTH1, two main ferritins responsible for iron storage, were upregulated in BALF of asthma mice. However, the iron level in lung tissue was greatly elevated in asthma mice, suggesting an excessive iron accumulation induced by asthma. The above iron metabolism in asthma mice was partly abolished by Ferr-1 and 3-MA. Additionally, studies have shown that when the levels of intracellular iron are low, it binds to FTH1 in autophagosomes and transfers autophagosomes to lysosomes to degrade ferritin and release free iron ion [32]. NCOA4, a cargo receptor mediating ferritinophagy, is responsible for the degradation of ferritin by autophagy, which is critical part contributing to cell ferroptosis [33, 34]. Our results revealed that the expression of the autophagy-marked protein (LC-3) was enhanced in lung tissues of asthma mice and bronchial epithelial cells stimulated with IL-13, and the expression of autophagy related proteins (NCOA4, TFR1, and DMT-1) in these cells and tissues were also promoted. However, the application of 3-MA abolished the changing of the expression of these proteins. Of note, the impacts of Ferr-1 on the expression of NCOA4, TFR1, and DMT-1 in asthma were weak, partly suggesting that ferritinophagy was not the core regulatory mechanism by which Ferr-1 suppressed ferroptosis to ameliorate asthma.

## 5. Conclusion

Above all, we detected the relationship between ferroptosis and the occurrence of asthma. According to the results, we can get the conclusion that Ferr-1 and 3-MA ameliorated ferroptosis in OVA-induced asthma model and in IL-13-challenged BEAS-2B cells, which provides a new strategy for the clinical treatment of asthma.

## Data Availability

All data generated in this study have been included in this article.

## Abbreviations

OVA: Ovalbumin
Ferr-1: Ferrostatin-1
3-MA: 3-Methyladenine
ALI: Air-liquid interface
FTL: Ferritin light chain
FTH1: Ferritin heavy chain
BALF: Bronchoalveolar lavage fluid
GSH: Reduced glutathione
GSSG: Oxidized glutathione
GPX4: Glutathione peroxidase 4
SLC7A11: Solute carrier family 7 member 11
SLC3A2: Solute carrier family 3 member 2
ALOX5: Arachidonate-5-lipoxygenase
NCOA4: Nuclear receptor coactivator 4
TFR1: Transferrin receptor
DMT-1: Divalent metal transporter 1
ATG5: Autophagy-related 5
ATG7: Autophagy-related 7
IREB2: Iron response element binding protein 2
ROS: Reactive oxygen species
SOD: Superoxide dismutase
MDA: Malonaldehyde.
